# Structure-based mechanism of action of a viral poly(ADP-ribose) polymerase 1-interacting protein facilitating virus replication

**DOI:** 10.1107/S2052252518013854

**Published:** 2018-10-31

**Authors:** Woo-Chang Chung, Junsoo Kim, Byung Chul Kim, Hye-Ri Kang, JongHyeon Son, Hosam Ki, Kwang Yeon Hwang, Moon Jung Song

**Affiliations:** a Virus–Host Interactions Laboratory, Department of Biosystems and Biotechnology, Korea University, 145 Anam-ro, Seongbuk-gu, Seoul 02841, Republic of Korea; b Structural Proteomics Laboratory, Department of Biosystems and Biotechnology, Korea University, 145 Anam-ro, Seongbuk-gu, Seoul 02841, Republic of Korea

**Keywords:** viral PARP-1-interacting protein, open reading frame 49, poly(ADP-ribose) polymerase 1, murine gammaherpesvirus 68, Kaposi’s sarcoma-associated herpesvirus, structure determination, X-ray crystallography

## Abstract

Poly(ADP-ribose) polymerase 1 (PARP-1), an enzyme that modifies nuclear proteins by poly(ADP-ribosyl)ation, regulates various cellular activities and restricts the lytic replication of oncogenic gammaherpesviruses by inhibiting the function of replication and transcription activator (RTA), a key switch molecule of the viral life cycle. A viral PARP-1-interacting protein (vPIP) encoded by murine gammaherpesvirus 68 (MHV-68) *orf49* facilitates lytic replication by disrupting interactions between PARP-1 and RTA. Here, the structure of MHV-68 vPIP was determined at 2.2 Å resolution. The structure consists of 12 α-helices with characteristic N-terminal β-strands (Nβ) and forms a V-shaped-twist dimer in the asymmetric unit. Structure-based mutagenesis revealed that Nβ and the α1 helix (residues 2–26) are essential for the nuclear localization and function of vPIP; three residues were then identified (Phe5, Ser12 and Thr16) that were critical for the function of vPIP and its interaction with PARP-1. A recombinant MHV-68 harboring mutations of these three residues showed severely attenuated viral replication both *in vitro* and *in vivo*. Moreover, ORF49 of Kaposi’s sarcoma-associated herpesvirus also directly interacted with PARP-1, indicating a conserved mechanism of action of vPIPs. The results elucidate the novel molecular mechanisms by which oncogenic gammaherpesviruses overcome repression by PARP-1 using vPIPs.

## Abstract 2

The structure of a viral poly(ADP-ribose) polymerase 1 (PARP-1)-interacting protein from murine gammaherpesvirus 68 (MHV-68) is reported. Structure-based mutagenesis revealed residues that are critical for function and interaction with PARP-1, and a recombinant MHV-68 harboring mutations of these three residues showed severely attenuated viral replication in infected cells and mice. A homologous vPIP protein of Kaposi’s sarcoma-associated herpesvirus also interacted with PAPR-1 and disrupted its inhibitory function, suggesting the conserved molecular mechanism of vPIPs to facilitate viral replication among gammaherpesviruses.

## 1. Introduction   

Poly(ADP-ribose) polymerase 1 (PARP-1) is a nuclear enzyme that catalyzes poly(ADP-ribosyl)ation (PARylation) of target proteins by transferring the ADP-ribose unit from NAD^+^ (Rouleau *et al.*, 2010 ▸). PARP-1 activity is involved in several important cellular functions such as differentiation, proliferation, malignant transformation and DNA damage repair (Kim *et al.*, 2005 ▸; Ko & Ren, 2012 ▸; Luo & Kraus, 2012 ▸; Gibson & Kraus, 2012 ▸). Of note, PARP-1 activity has been implicated in virus–host conflicts either positively or negatively (Gupte *et al.*, 2017 ▸; Ko & Ren, 2011 ▸, 2012 ▸; Rom *et al.*, 2015 ▸; Dandri *et al.*, 2002 ▸). For example, the replication of retroviruses such as human immunodeficiency virus 1 and human T-lymphotropic virus 1 is affected by PARP-1 activity at the steps of genome integration, genome replication and viral gene transcription (Zhang *et al.*, 2002 ▸; Kameoka *et al.*, 1999 ▸, 2005 ▸; Bueno *et al.*, 2013 ▸; Ha *et al.*, 2001 ▸; Rom *et al.*, 2015 ▸). PARP-1 modulates hepatitis B virus replication and DNA integration into the host chromosome (Ko & Ren, 2011 ▸; Dandri *et al.*, 2002 ▸). PARP-1 is also associated with herpesvirus replication; herpes simplex virus 1 activates PARP-1 during replication (Grady *et al.*, 2012 ▸).

The roles of PARP-1 in oncogenic gammaherpesviruses, including Epstein–Barr virus (EBV), Kaposi’s sarcoma-associated herpesvirus (KSHV) and murine gammaherpes­virus 68 (MHV-68), have been studied in more detail and involve the suppression of lytic replication and reactivation (Gwack *et al.*, 2003 ▸; Ohsaki *et al.*, 2004 ▸; Wang *et al.*, 2008 ▸; Lupey-Green *et al.*, 2017 ▸; Mattiussi *et al.*, 2007 ▸; Martin *et al.*, 2016 ▸; Tempera *et al.*, 2010 ▸). As a key switch molecule of the gammaherpesvirus life cycle, a protein called replication and transcription activator (RTA) performs an essential function in lytic replication and reactivation from latency (Sun *et al.*, 1998 ▸; Lukac *et al.*, 1998 ▸, 1999 ▸). In gamma-2 herpesviruses (rhadinoviruses), including KSHV and MHV-68, PARP-1 PARylates RTA and inhibits its transactivation, resulting in the overall suppression of viral lytic replication (Gwack *et al.*, 2003 ▸). In EBV, a gamma-1 herpesvirus (lymphocryptovirus), the binding of PARP-1 to a lytic promoter also represses viral reactivation (Lupey-Green *et al.*, 2017 ▸).

Meanwhile, gammaherpesviruses encode viral proteins to overcome negative regulation by PARP-1; for example, viral processivity factors in KSHV and MHV-68 induce the degradation of PARP-1 in a proteasome-dependent manner, thus promoting lytic replication (Cheong *et al.*, 2015 ▸). In MHV-68, ORF49, a tegument protein, facilitates RTA-mediated transactivation by interacting with PARP-1, thereby disrupting interactions between RTA and PARP-1 (Lee *et al.*, 2007 ▸; Noh *et al.*, 2012 ▸). ORF49 homologs from human gammaherpes­viruses such as Epstein–Barr virus BRRF1 (also called Na) and KSHV ORF49 have been reported to promote viral lytic replication by cooperating with RTA (González *et al.*, 2006 ▸; Hong *et al.*, 2004 ▸), although it is not known whether they share the molecular mechanisms of PARP-1 interaction and inhibition. Yeast two-hybrid screening of EBV viral proteins using a human cDNA library revealed that EBV BRRF1 interacts with PARP-4, a homolog of PARP-1 (Calderwood *et al.*, 2007 ▸), while the direct inhibition of RTA by PARP-1 has not been reported.

Here, we show a direct physical interaction of MHV-68 ORF49 with PARP-1 in solution and propose calling this protein viral PARP-1-interacting protein (vPIP). We determined the X-ray crystal structure of vPIP at 2.2 Å resolution. The vPIP protein consists of 12 α-helices and two β-strands and forms a V-shaped-twist dimer in the asymmetric unit. We performed structure-based mutagenesis to identify domains and residues of vPIP that are crucial for subcellular localization, protein–protein interactions and the functional activity of vPIP. The functional significance of crucial residues was next examined in the context of virus replication both *in vitro* and *in vivo* using a recombinant virus harboring the mutations. Finally, the protein encoded by KSHV *orf49* (ORF49_KSHV_) was found to interact with PARP-1, thereby relieving PARP-1 repression of RTA. Based on the structural information, this study highlights the conserved molecular mechanism by which vPIPs of oncogenic gammaherpesviruses facilitate viral replication *in vitro* and *in vivo*.

## 2. Materials and methods   

### 2.1. Purification of proteins in bacteria   

The genomic regions for MHV-68 vPIP and ORF49_KSHV_ were cloned into pET-22b or pET-28a plasmids (Novagen), respectively, using gene-specific primers (Supplementary Table S1). MHV-68 vPIP and ORF49_KSHV_ were overexpressed in *Escherichia coli* Rosetta 1 strain and BL21 strain (Novagen) at 18°C after induction with 0.5 m*M* isopropyl β-d-1-thio­galactopyranoside (IPTG). The proteins were purified by Ni–NTA affinity chromatography. A linear concentration gradient was applied to elute the product at a flow rate of 5 ml min^−1^ in a buffer consisting of 50 m*M* HEPES pH 7.5, 150 m*M* NaCl, 5 m*M* β-mercaptoethanol, 500 m*M* imidazole. The proteins were further purified by ion-exchange chromatography with a linear NaCl gradient and were concentrated using Amicon Ultra centrifugal filters (Merck Millipore). A size-exclusion chromatography step was next performed on a Superdex 200 26/60 column (GE Healthcare) equilibrated with final buffer (50 m*M* HEPES pH 7.5, 100 m*M* NaCl, 1% glycerol, 10 m*M* dithiothreitol). Finally, the proteins were concentrated to 15 mg ml^−1^ for crystallization and surface plasmon resonance analysis using Amicon Ultra centrifugal filters and stored at −80°C.

### 2.2. Crystallization   

Crystals were grown using a sitting-drop vapor-diffusion screen in which 0.5 µl protein sample was mixed with an equal volume of screening solution from the Crystal Screen kit in 96-well Intelli-Plates (Hampton Research) and using standard hanging-drop vapor-diffusion techniques. An initial crystallization hit was found in a saturating solution of 0.1 *M* Tris–HCl pH 8.2, 0.33 *M* sodium/potassium tartrate, 0.5% polyethylene glycol 5000 monomethyl ether. Crystals were obtained by mixing 1 µl protein solution with 1 µl reservoir solution. The crystals were transferred into reservoir solution containing 20% ethylene glycol before flash-cooling in liquid nitrogen.

### 2.3. Structure determination   

Diffraction data were collected on beamline BL1A at KEK, Photon Factory, Japan and the data were processed using *SCALEPACK* and *DENZO* from the *HKL*-2000 software package. The crystal belonged to space group *P*3_2_21, with unit-cell parameters *a* = *b* = 134.179, *c* = 157.158 Å, α = β = 90, γ = 120°. There are two molecules in the asymmetric unit. Single-wavelength anomalous dispersion (SAD) data were collected from selenomethionine-labeled vPIP crystals at an inflection wavelength of 0.9792 Å and were processed using *HKL*-2000. The *PHENIX AutoSol* program was used for phasing (Adams *et al.*, 2010 ▸). The initial model was manually rebuilt in *Coot* and refined using *CCP*4*i* (Winn *et al.*, 2011 ▸). The final refinement was conducted using *phenix.refine* in *PHENIX*. The final model had *R*_work_ = 23.6% and *R*_free_ = 27.3%. The description of the crystal structure was prepared in *PyMOL* (DeLano, 2001 ▸). Data-collection and refinement statistics are summarized in Supplementary Table S2.

### 2.4. Multi-angle light-scattering assay   

Proteins in 50 m*M* HEPES pH 7.5 with 100 m*M* NaCl were studied by analytical size-exclusion chromatography on a WTC-050S5 column (Wyatt Technology) and directly flowed into a Wyatt DAWN HELEOS II light-scattering detector and a Wyatt Optilab T-rEX refractive-index detector (Wyatt Technology). The column was employed to determine the average molecular mass of the elution peak from the Rayleigh scattering intensity as a function of the scattering index (LSR) and the buffer scattering index (dRI) using *ASTRA* 6 (Wyatt Technologies) (Trathnigg, 1995 ▸).

### 2.5. Surface plasmon resonance (SPR) binding assays   

SPR assays were conducted on a Biacore T-100 instrument (GE Healthcare). To measure interactions between PARP-1 and vPIP, the surface of the sensor chip CM5 (GE Healthcare) has a carboxymethylated dextran matrix covalently attached to a surface coating on a gold film. Kinetic analysis was carried out at a flow rate of 30 µl min^−1^. The standard running buffer was HBS-EP [10 m*M* HEPES pH 7.4, 150 m*M* NaCl, 3 m*M* EDTA, 0.005%(*v*/*v*) surfactant P20; GE Healthcare]. The results were processed using the Biacore T-100 analysis software. His-tagged mouse PARP-1 protein (Sino Biological) was reconstituted in a sterile buffer consisting of 20 m*M* Tris pH 8.0, 500 m*M* NaCl, 10% glycerol, 0.1 m*M* tris(2-carboxyethyl)phosphine hydrochloride. Capturing the purified His-tagged mouse PARP-1 protein in flow cell 2 was performed by injecting a 200 µg ml^−1^ protein solution for 1 h at a flow rate of 5 µl min^−1^. Flow cell 1 served as a reference for the substrate in terms of nonspecific binding, drift and the bulk refractive index. Compounds were assayed in single-cycle kinetics mode in five-point and six-point twofold concentration series from 0.1 to 3.45 µ*M* for MHV-68 vPIP and from 0.22 to 7.12 µ*M* for ORF49_KSHV_. Data were processed and fitted to a 1:1 binding model in the Biacore T100 evaluation software to determine the binding kinetic rate constants *k*_a_ (on rate) and *k*_d_ (off rate), and the equilibrium dissociation constant *K*_d_.

### 2.6. Cell culture and virus   

HEK293T, HeLa, BHK21 and Vero cells were cultured in complete Dulbecco’s modified Eagle’s medium (HyClone) containing 10% fetal bovine serum (FBS; HyClone) and supplemented with 100 U ml^−1^ penicillin and 100 µg ml^−1^ streptomycin (HyClone). The MHV-68 virus was originally purchased from the American Type Culture Collection (ATCC; catalog No. VR1465). The amplified or reconstituted viruses were titrated by plaque assays on Vero cells overlaid with 1% methylcellulose (Sigma) in the normal growth medium.

### 2.7. Plasmid   

MHV-68 vPIP mutant constructs were cloned into the pENTR vector (Invitrogen) using the primers listed in Supplementary Table S1. The entry clones were further transferred to the desired destination vectors containing additional sequences to generate MYC-tagged, FLAG-tagged or GFP-tagged vPIP mutants using the Gateway technology (Invitrogen). FLAG-tagged vPIPΔN+NLS_SV40_ was generated by inserting a classical nuclear localization signal (NLS) from the SV40 large T-antigen (PKKKRKV; Ng *et al.*, 2018 ▸). A PARP-1 expression construct (pCMV5-PARP-1) was a kind gift from Dr W. Lee Kraus at the University of Texas Southwestern Medical Center (Dallas, Texas, USA). FLAG-tagged and GFP-KSHV ORF49 constructs were generated as described previously (Chung *et al.*, 2015 ▸).

### 2.8. Luciferase reporter assays   

The Luciferase Reporter Assay Kit (Promega) was applied to measure the activity of the RTA promoter (Rp-LUC) or the RTA-responsive promoter (M3p-LUC) (Lukac *et al.*, 1999 ▸; Chung *et al.*, 2015 ▸). To test the functionality of vPIP mutants in terms of the promoter activity, HEK293T cells were transfected using polyethylenimine (1 mg ml^−1^; Sigma) with a reporter construct, an RTA expression plasmid, a β-galactosidase (β-gal) expression plasmid and a vPIP mutant plasmid, as described previously (Boussif *et al.*, 1995 ▸). 26 h post-transfection, the cells were harvested and analyzed by the luciferase reporter assays according to the manufacturer’s instructions. Each transfection for the reporter assays was performed in triplicate. In all of the assays, luminescence from the reporters was normalized to the activity of β-galactosidase.

### 2.9. Creation of the MHV-68 vPIP mI and vPIP mI-MR recombinant viruses   

The recombinant MHV-68 BAC plasmids expressing the vPIP mI and vPIP mI-MR viruses were generated by a RED-mediated recombination method from BAC-containing *E. coli* cells (GS1783; Tischer *et al.*, 2006 ▸; Yu *et al.*, 2000 ▸). Briefly, we generated PCR template fragments containing kanamycin-I-SceI from pEntransposon-KanR (STM vector, Finnzyme) with the forward primer 5′-AAGCCACGTTGTGTC-3′ and the reverse primer 5′-ATTACCCTGTTATCCCTATTTTCGACCGAATAAAG-3′. Electroporation of the kanamycin-I-SceI-containing PCR fragment was used to transfect GS1783 cells. After a selection procedure, positive clones were confirmed by PCR screening and sequencing. To excise the virus genome from the BAC sequence, vPIP mI or vPIP mI-MR BAC DNA was transfected using Lipofectamine Plus (Invitrogen) with a Cre expression plasmid into BHK21 cells (5 × 10^5^) in six-well culture plates. The genome integrity of the reconstituted virus was verified by restriction-enzyme digestion.

### 2.10. Quantitative real-time PCR   

Infected BHK21 cells, homogenized lung samples or spleno­cytes from infected mice were lysed overnight in a buffer consisting of 20 m*M* Tris–HCl pH 7.5, 10 m*M* EDTA, 100 m*M* NaCl, 0.5% SDS with 500 µg ml^−1^ proteinase K, and viral genomic DNAs were isolated by the phenol:chloroform:isoamyl alcohol [25:24:1(*v*:*v*:*v*)] extraction method. M1 locus-specific primers (forward, 5′-CCTGGCCATGGTTACATACTC-3′; reverse, 5′-GGAACATAATCCATAAGCAGGGT-3′) were used to determine the copy numbers of viral genomic DNAs (Rickabaugh *et al.*, 2005 ▸). Real-time PCR was carried out on a Rotor-Gene Q real-time PCR detection system (Qiagen, Venlo, Netherlands). Real-time PCR with SYBR Green was run at 95°C for 15 min, followed by 45 cycles of 95°C for 10 s, 55°C for 15 s and 72°C for 20 s.

### 2.11. Western blot analysis   

The whole-cell lysates were resolved by SDS–PAGE, transferred to a polyvinylidene fluoride membrane and probed with primary antibodies against FLAG-M2 (1:2000; Sigma), MYC (1:2000; made in our laboratory), ORF45 (1:500; made in our laboratory), GFP (1:500; Santa Cruz Biotechnology), PARP-1 (1:1000, BD Biosciences) or α-tubulin (1:2000; Sigma). A goat anti-rabbit or goat anti-mouse immunoglobulin G antibody conjugated with horseradish peroxide (a secondary antibody; Santa Cruz Biotechnology) was detected using ECL plus Western blotting detection reagents (ELPIS), and the signals were analyzed on LAS-4000, a chemiluminescent image analyzer (Fujifilm).

### 2.12. Immunoprecipitation (IP) assays   

After 48 h of transfection, HEK293T cells were harvested with IP buffer consisting of 20 m*M* HEPES pH 7.4, 100 m*M* NaCl, 0.5% Nonidet P-40, 1% Triton X-100 supplemented with a 1% volume of a protease-inhibitor cocktail (Sigma). The cell lysates were rotated at 4°C for 1 h and cell debris was removed by centrifugation (14 000*g*, 4°C, 10 min). An anti-FLAG antibody (M2, Sigma) was added and the lysates were incubated at 4°C with rotation. 30 µl of Protein A/G Agarose beads (Pierce) were then added and incubation was continued for 16 h at 4°C. The beads were washed with IP buffer and the proteins were analyzed by Western blotting.

### 2.13. Immunofluorescence assays (IFAs) and confocal microscopy   

After 24 h, the transfected HEK293T or HeLa cells were fixed for 15 min with 4% paraformaldehyde and 0.15% picric acid in PBS. The blocking step was performed with 10% normal goat serum in PBS containing 0.3% Triton X-100 and 0.1% BSA. The anti-PARP-1 antibody (Cell Signaling Technology) was incubated with the cells as a primary antibody for 16 h at 4°C. A rabbit anti-Rho antibody (Jackson Immuno­Research) was incubated with the cells as a secondary antibody for 45 min at room temperature. 4′,6-Diamidino-2-phenyl­indole (DAPI; 1:1,000) was used for nucleus staining for 3 min at room temperature. The fluorescent images were captured at a magnification of 1000× using a confocal laser scanning microscope (LSM 5 Exciter, Zeiss).

### 2.14. Mouse experiments   

All animal experiments were approved by the Korea University Institutional Animal Care and Use Committee (KUIACUC-2016-119) in accordance with institutional guidelines. Six-week-old BALB/c mice (Samtako) were intranasally infected with WT, vPIP-ST, vPIP mI or vPIP mI-MR virus (1000 PFU per mouse, *n* = 5 in each group) under anesthesia. The mice were euthanized 6 d post-infection during acute infection and 17–18 d post-infection during latent infection. For acute-infection analysis, the lung tissues were homogenized in 1 ml DMEM and the virus titers were determined by plaque assays. The viral genome loads in the lung tissues were measured by quantitative real-time PCR. For quantification of latent viral loads, *ex vivo* limiting-dilution assays and infectious-center assays were performed on Vero cells as described elsewhere (Lee *et al.*, 2007 ▸; Noh *et al.*, 2012 ▸).

## 3. Results   

### 3.1. Overall characterization of vPIP   

**Figure 1:**
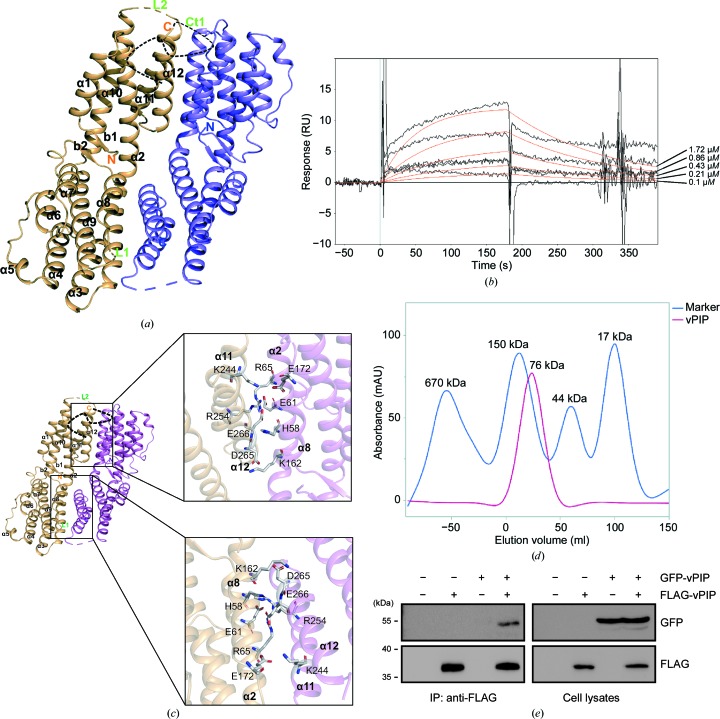
Overall structure and characterization of MHV-68 vPIP. (*a*) Crystal structure representation of MHV-68 vPIP. vPIP forms a dimer in the asymmetric unit with space group *P*3_2_21. The crystal structure is shown with secondary structure that includes 12 α-helices and two β-strands. The dotted lines indicate the disordered loops (L1, L2 and Ct1). The chain *A* helix bundle is shown in light orange and the chain *B* helix bundle is shown in violet. (*b*) SPR analysis of vPIP with PARP-1. The vPIP protein was injected at five concentrations (1.72, 0.86, 0.43, 0.21 and 0.1 µ*M*). Dissociation data were collected for 120 s. Black lines show the actual data; the orange lines are curve fits. (*c*) A structural model of the dimer interface of vPIP. Residues in the dimer interface are presented as stick models. In the upper and lower panels each residue in chain *A* (His58, Glu61, Arg65, Lys162, Glu172, Lys244, Arg254, Asp265 and Glu266) and in chain *B* (His58, Glu61, Arg65, Lys162, Arg72, Glu172, Lys244, Arg254, Asp265 and Glu266) involved in hydrogen bonds and salt bridges is indicated. The interaction of residues was calculated using *PISA*. (*d*) Multi-angle light-scattering and refractive-index curves for vPIP dimerization. Light scattering (LS) is shown in blue and the differential refractive index (dRI) is shown in red. The buffer was removed and the LS and refractive index were measured and plotted against the protein sample. (*e*) GFP-tagged vPIP and FLAG-tagged vPIP were transfected into HEK293T cells for 48 h. The cells were harvested and subjected to co-IP assays using anti-FLAG. The results were analyzed by Western blotting.

We determined the structure of full-length MHV-68 ORF49 (PDB entry 6a4v; later called vPIP) at 2.2 Å resolution (Fig. 1 ▸ *a*). The structure consists of 12 α-helices and characteristic N-terminal β-strands (Nβ) and forms a V-shaped-twist dimer in the asymmetric unit. Three regions, L1 (amino-acid residues 175–176), L2 (amino-acid residues 231–237) and Ct1 (amino-acid residues 280–301), were not visible in the electron-density map of the crystal, suggesting that these regions have high flexibility. In our previous report, MHV-68 ORF49 was shown to interact with PARP-1 in a cellular environment (Noh *et al.*, 2012 ▸). The results of surface plasmon resonance (SPR) analysis of the purified ORF49 protein regarding PARP-1 binding affinity *in vitro* indicated that *K*_d_ is approximately 930 n*M*: sufficient affinity for PARP-1 (Fig. 1 ▸ *b*). This result clearly revealed the direct physical interaction of ORF49 and PARP-1; therefore, we propose calling MHV-68 ORF49 a viral PARP-1-interacting protein (vPIP). The structure of vPIP was found to have a dimer in the asymmetric unit, and its interface is formed by hydrogen bonds and salt bridges (Fig. 1 ▸ *c*). The salt bridges are Arg65_*A*_–Glu266_*B*_ and Lys162_*A*_–Asp265_*B*_ (the protomer is shown as a subscript). Size-exclusion chromatography with multi-angle light scattering (SEC-MALS) verified vPIP dimerization *in vitro* because the molecular weight (MW) was found to be approximately 76 kDa: double the molecular weight of the vPIP monomer (38 kDa; Fig. 1 ▸ *d*). Co-immunoprecipitation (co-IP) with an anti-FLAG antibody confirmed the dimerization of vPIP in HEK293T cells co-transfected with FLAG-tagged vPIP and GFP-fused vPIP (Fig. 1 ▸ *e*). These results indicate that vPIP exists as a dimer both in solution and in the cell.

### 3.2. Mutagenesis and subcellular localization of vPIP mutants   

**Figure 2:**
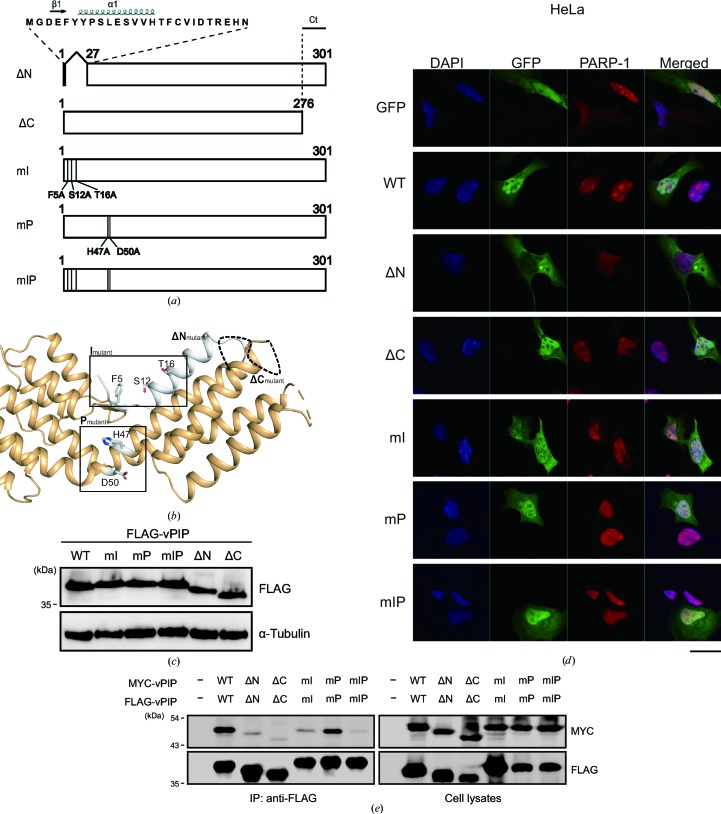
Construction and characterization of vPIP mutants. (*a*) Schematic diagram of vPIP deletion and point mutants. Two deletion mutants, vPIP ΔN (deletion of amino acids 2–26) for Nβ and α1 and vPIP ΔC (deletion of amino acids 277–301), and two point mutants, vPIP mI (F5A, S12A and T16A) for interaction sites and vPIP mP (H47A and D50A) for pore sites, were constructed. An additional mutant containing both mI and mP was also generated (vPIP mIP). (*b*) The structure of vPIP mutations with positional indicators. The positions of the mutations are marked in the monomer structure of vPIP. (*c*) Expression of vPIP mutants. FLAG-tagged vPIP mutant constructs were transfected into HeLa cells. After 24 h, expression of the vPIP mutants was analyzed by Western blotting. (*d*) Subcellular localization of vPIP mutants. HeLa cells were transfected with the GFP-tagged vPIP mutants, fixed at 24 h post-transfection and immunostained with anti-PARP-1 antibody. The nuclei were stained with DAPI (blue). The scale bar is 20 µm in length. (*e*) Dimerization of vPIP mutants. MYC-tagged vPIP mutants were co-transfected with FLAG-tagged vPIP mutants into HEK293T cells for 48 h. The cells were harvested and subjected to co-IP assays using anti-FLAG. The results were analyzed by Western blotting.

On the basis of the structural information on vPIP, we constructed two deletion mutants: vPIP ΔN (deletion of amino acids 2–26) lacking the N-terminal β1 and α1, and vPIP ΔC (deletion of amino acids 277–301) lacking the C-terminal Ct1. In addition, two point mutants, vPIP mI (F5A, S12A and T16A) and vPIP mP (H47A and D50A), were constructed to understand the functional importance of some vPIP regions. Three residues (Phe5, Ser12 and Thr16), which were found on the surface of vPIP and are likely to participate in interactions with other proteins, were substituted by alanines to create vPIP mI (an interacting-site mutant), whereas two residues (His47 and Asp50) at the center of the dimer that form a cavity were substituted by alanines to create vPIP mP (a pore-site mutant; Figs. 2 ▸ *a* and 2 ▸ *b*). A mutant containing mutations at both the interaction site and the pore site was also generated (vPIP mIP). All of the constructed mutants showed a normal level of expression comparable to that of wild-type (WT) vPIP in transfected cells (Fig. 2 ▸ *c*). To examine the subcellular localization of these mutants, GFP-fused mutant constructs were transfected into HeLa cells. Although the WT vPIP protein was expressed in both the cytoplasm and the nucleus, as previously reported (Lee *et al.*, 2007 ▸), the vPIP ΔN mutant localized only to the cytoplasm; however, the other mutants were expressed both in the nucleus and the cytoplasm (Fig. 2 ▸ *d*). The localization patterns of the mutants in HEK293T cells were consistent with the above findings (Supplementary Fig. S1). These results suggest that the N-terminus of vPIP may be essential for nuclear localization of the vPIP protein. When the ability of vPIP mutants to form a dimer was determined in HEK293T cells transfected with FLAG-tagged and MYC-tagged vPIP mutants in a co-IP experiment, all of the mutants were defective in dimerization except for the pore-site mutant (vPIP mP; Fig. 2 ▸ *e*).

### 3.3. Effects of vPIP mutations on the regulation of viral lytic replication   

**Figure 3:**
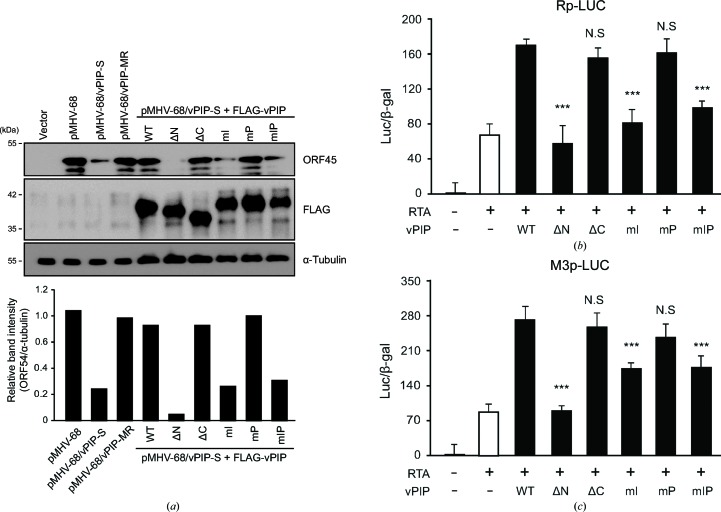
Effects of vPIP mutations on the regulation of virus lytic replication. (*a*) Trans-complementation of vPIP-S virus replication by vPIP mutants. Vero cells were co-transfected with FLAG-tagged vPIP mutants in the presence of the vPIP-S virus BAC DNA (pMHV-68/vPIP-S). MHV-68 BAC DNA (pMHV-68) and vPIP-S-MR were used as a control. After 5 d of transfection, the cell lysates were subjected to Western blotting. The band intensity of the ORF45 protein was measured and normalized with that of α-tubulin using the *ImageJ* program. (*b*, *c*) The effect of vPIP mutants on RTA-mediated transactivation. HEK293T cells were transfected with the reporter construct containing RTA promoter (Rp-LUC) (*b*) or M3 promoter (M3p-LUC) (*c*), and vPIP mutants in the presence of RTA-expressing plasmid. Each transfection was performed in triplicate, with a β-galactosidase-expressing plasmid included as an internal control. Statistical analysis was performed using a two-sided Student’s t-test (*** denotes a *P* value of <0.005 against WT vPIP expressing samples).

MHV-68 vPIP facilitates viral lytic replication by co­operating with RTA (Lee *et al.*, 2007 ▸). To test the functionality of the constructed mutants, we determined whether each vPIP mutant can trans-complement the highly attenuated replication phenotype of a vPIP-deficient virus (Fig. 3 ▸ *a*). The mutant vPIP constructs were co-transfected with BAC DNA harboring the entire genome of MHV-68 (pMHV-68) with triple stop codons in the *orf49* (vPIP) gene (vPIP-S; Noh *et al.*, 2012 ▸). BAC DNA carrying recombinant virus vPIP-MR, which is free of the triple stop codons of vPIP-S, as well as the wild-type pMHV-68 BAC DNA, served as controls. The results showed that the vPIP ΔN, vPIP mI and vPIP mIP proteins failed to reverse the attenuation of replication in the vPIP-S virus, whereas vPIP ΔC and vPIP mP were capable of trans-complementing the replication defect of the vPIP-S virus (Fig. 3 ▸ *a*). Because vPIP enhances RTA-mediated transactivation of lytic gene promoters (Lee *et al.*, 2007 ▸), we tested whether vPIP mutants retained the ability to increase RTA-mediated transactivation in reporter assays with the RTA promoter (Rp-LUC) or RTA-responsive M3 promoter (M3p-LUC; Figs. 3 ▸ *b* and 3 ▸ *c*). In agreement with other reports (Noh *et al.*, 2012 ▸; Lee *et al.*, 2007 ▸), RTA alone activated both promoters and WT vPIP enhanced RTA-mediated trans­activation (Figs. 3 ▸ *b* and 3 ▸ *c*). In contrast, the mutant proteins vPIP ΔN, vPIP mI and vPIP mIP were unable to promote RTA-mediated transactivation, whereas vPIP ΔC and vPIP mP enhanced it to levels similar to those of the WT (Figs. 3 ▸ *b* and 3 ▸ *c*). These results indicate that the N-terminus of vPIP is essential for its function, as shown for vPIP ΔN. Moreover, the N-terminal residues, especially Phe5, Ser12 and Thr16, of vPIP were critical for the enhancement of RTA-mediated trans­activation in the nucleus.

### 3.4. Molecular interactions of vPIP mutants with PARP-1 and RTA   

**Figure 4:**
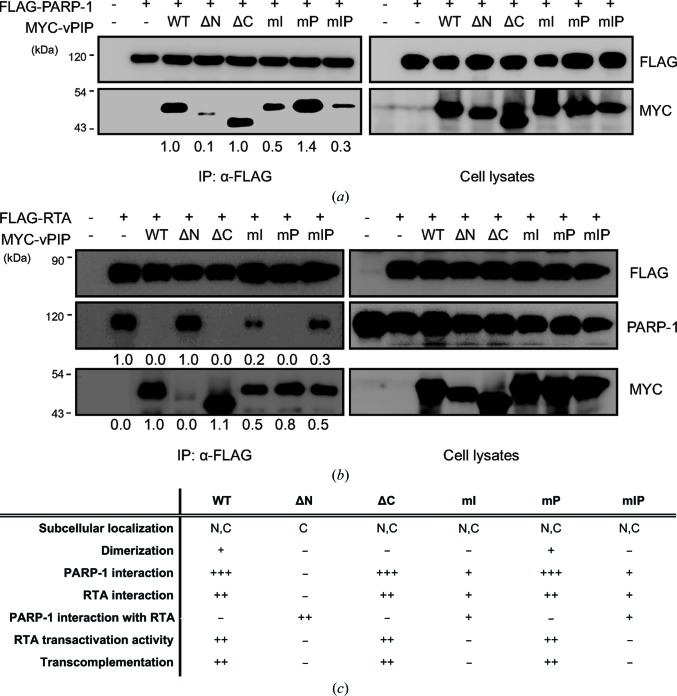
Molecular interactions of vPIP mutants with PARP-1 or RTA. (*a*) Interaction between vPIP mutants and PARP-1. MYC-tagged vPIP mutants were co-transfected with FLAG-tagged PARP-1 into HEK293T cells. The cells were harvested 48 h post-transfection and analyzed by co-IP assays with the anti-FLAG antibody. The results were examined by Western blotting. The band intensity of MYC in IP blots was measured using the* ImageJ* software. (*b*) Inhibition of interactions between RTA and PARP-1 by vPIP mutants. MYC-tagged vPIP mutants were co-transfected with FLAG-tagged RTA into HEK293T cells. The cells were harvested 48 h post-transfection and subjected to co-IP assays with the anti-FLAG antibody. The results were examined by Western blotting. The band intensity of PARP-1 and MYC in IP blots was measured in *ImageJ*. (*c*) A summary of subcellular localization, protein–protein interactions and functional activities of WT vPIP and mutants.

As a molecular mechanism for the promotion of viral lytic replication by vPIP, it has been proposed that vPIP interacts with and sequesters PARP-1, thus disrupting the interaction of PARP-1 with RTA and reducing the amount of PARylated RTA (Noh *et al.*, 2012 ▸). In addition, vPIP directly binds to RTA, suggesting that there may be a direct effect of vPIP on the activity of RTA (Noh *et al.*, 2012 ▸). Firstly, we examined the molecular interaction of vPIP mutants with PARP-1 by co-transfecting FLAG-PARP-1 with one of the MYC-vPIP mutants followed by a co-IP experiment (Fig. 4 ▸ *a*). Consistent with the functional results on vPIP mutants in trans-complementation and reporter assays, the inter­actions of the mutant proteins vPIP ΔN, vPIP mI and vPIP mIP with PARP-1 were severely impaired or atten­uated, but those of the mutant proteins vPIP ΔC and vPIP mP were not affected. Next, we evaluated the effects of these mutations on the interaction of RTA with vPIP as well as that of RTA with PARP-1 (Fig. 4 ▸ *b*). Although the interactions of the mutant proteins vPIP ΔC and vPIP mP with RTA showed no significant difference from that of the WT, there were reduced or nonexistent interactions of vPIP ΔN, vPIP mI and vPIP mIP with RTA. Moreover, the intensity of the RTA–PARP-1 interaction was affected by the presence of vPIP mutant proteins; the vPIP ΔC and vPIP mP mutants completely disrupted the RTA–PARP-1 interaction, as did the WT, whereas vPIP ΔN did not disrupt the RTA–PARP-1 interaction at all, and the vPIP mI and vPIP mIP mutant proteins were partially impaired in interfering with the RTA–PARP-1 inter­action (Fig. 4 ▸ *b*). To further examine whether the defective function of vPIP ΔN is owing to its cytoplasmic localization, an additional mutant construct, vPIPΔN+NLS_SV40_, was generated by inserting a classical nuclear localization signal (NLS) from the SV40 large T-antigen (PKKKRKV; Ng *et al.*, 2018 ▸). While the new construct was only expressed in the nucleus, it was still defective in interacting with PARP-1 and RTA like vPIP ΔN, thereby not being able to alleviate the repression of RTA by PARP-1 (Supplementary Fig. S2). Taken together, these results suggest that the N-terminal residues, especially Phe5, Ser12 and Thr16, of vPIP may be critical for the interaction of vPIP with PARP-1 and RTA, thereby interfering with the interactions between RTA and PARP-1. The molecular phenotypes of vPIP mutants are summarized in Fig. 4 ▸(*c*).

### 3.5. Construction and *in vitro* replication of the mutant virus (vPIP mI)   

**Figure 5:**
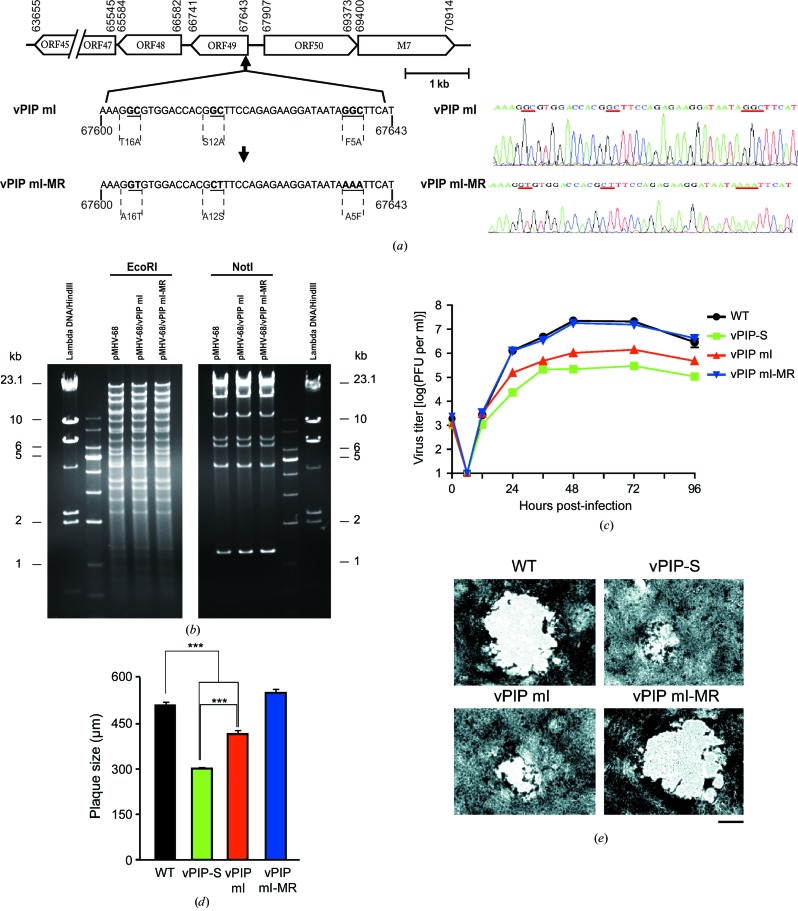
Creation and *in vitro* replication of the mutant virus (vPIP mI). (*a*) A schematic diagram of the ORF49 gene locus in the MHV-68 genome. Open reading frames are shown as boxes. The arrowheads of the boxes indicate the direction of transcription. The numbers indicate the positions of each part within the viral genome. The recombinant viruses vPIP mI and vPIP mI-MR were constructed as indicated and the mutated positions in the recombinant viruses were confirmed by sequencing. (*b*) The genome integrity of the recombinant virus BAC clones was verified by digestion with the EcoRI or NotI restriction enzyme. (*c*) Multiple-step replication curves of the WT, vPIP-S, vPIP mI and vPIP mI-MR viruses. BHK21 cells were infected with the WT, vPIP-S, vPIP mI or vPIP mI-MR virus in triplicate at a multiplicity of infection (MOI) of 0.05 and were harvested at the indicated time points. The virus titers in the cells and the supernatants were analyzed by plaque assays. (*d*, *e*) Plaque sizes of the WT, vPIP-S, vPIP mI and vPIP mI-MR viruses. Plaque assays were performed on Vero cells and the diameters of the plaques were determined for at least 100 plaques per virus. The average plaque sizes are shown with the standard error in (*d*). Statistical analysis was performed by a two-sided Student’s t-test (*** denotes *P* < 0.005). Representative pictures of actual plaques are shown in (*e*). The scale bar is 200 µm in length.

To investigate the roles of key residues (Phe5, Ser12 and Thr16) of vPIP in the context of virus replication, the vPIP mI recombinant virus containing alanine substitutions of three residues (F5A, S12A and T16A) was generated by a RED-mediated recombination method (Yu *et al.*, 2000 ▸; Tischer *et al.*, 2006 ▸). A corresponding marker rescue virus (vPIP mI-MR) that was free of these mutations was also constructed as a control (Fig. 5 ▸ *a*). The introduced mutations were confirmed by sequencing (Fig. 5 ▸ *a*), and the genome integrity of the recombinant virus clones was verified by restriction-enzyme digestion of the BAC clones (Fig. 5 ▸ *b*). In a multiple-step growth analysis of the WT, vPIP-S, vPIP mI and vPIP mI-MR viruses, the replication rates of the vPIP-S and vPIP mI viruses were found to be significantly attenuated when compared with those of the WT or vPIP mI-MR viruses (Fig. 5 ▸ *c*). Infection with the vPIP mI or vPIP-S virus resulted in plaques of smaller size relative to the WT and vPIP mI-MR viruses, although the plaques of the vPIP-S virus were even smaller than those of the vPIP mI virus (Figs. 5 ▸ *d* and 5 ▸ *e*). These results suggest that the three amino-acid residues at the N-terminus of vPIP that are important for its interaction with PARP-1 and derepression of RTA may be crucial for viral replication *in vitro*.

### 3.6. Acute infection and *in vivo* latency of the mutant virus (vPIP mI)   

**Figure 6:**
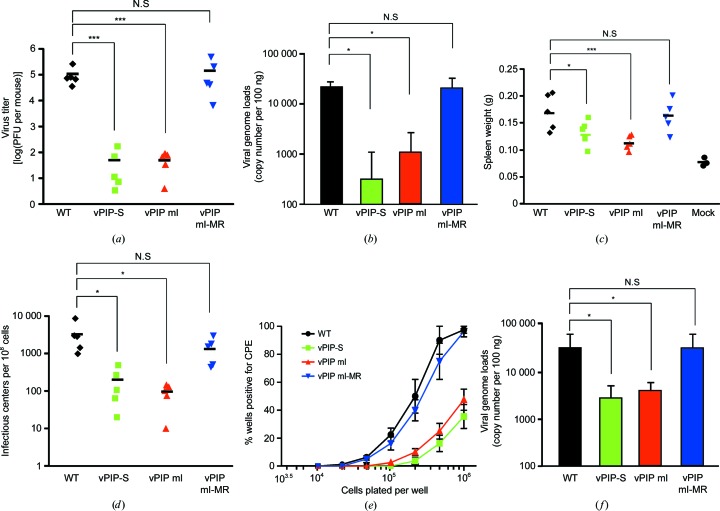
*In vivo* acute and latent infections by the mutant virus (vPIP mI). BALB/c mice were intranasally infected with 1000 plaque-forming units of WT, vPIP-S, vPIP mI or vPIP mI-MR virus. (*a*, *b*) Acute infection with the vPIP mI mutant in the lungs. At 6 d post-infection the lungs were excised and homogenized to determine lytic viral titers in plaque assays (*a*). Each symbol represents the viral titer from the lung tissue of individual mice, with the bar showing the mean value (*n* = 5 in each group). The genomic DNA samples were extracted from the lungs of infected mice. The viral genome copy numbers were quantitated by real-time PCR (*b*). The average viral genome copy numbers are shown with the standard error of the mean. (*c*–*f*) Latent infection with the mutant (vPIP mI) in the spleen. At 17–18 d post-infection, the spleens were excised and their weights were measured (*c*). The splenocytes were prepared and examined for the frequency of cells reactivating the virus by *ex vivo* infectious-center assays (*d*) and a limiting-dilution assay (*e*). In the limiting-dilution assay, each symbol represents the average percentage of wells positive for cytopathic effects with the standard error of the mean (*n* = 5 in each group). In infectious-center assays, each symbol represents the viral titer from individual mice, with the bar showing the mean value. The genomic DNA samples were extracted from splenocytes of the infected mice. The viral genome copy numbers were quantitated by real-time PCR (*f*). The average viral genome copy numbers are shown with standard errors of the mean (*n* = 5 in each group). Statistical analysis was performed by a two-sided Student’s t-test (* denotes *P* < 0.05 and *** denotes *P* < 0.005).

To determine the effect of vPIP mutations on MHV-68 infection *in vivo*, we intranasally infected BALB/c mice with WT, vPIP-S, vPIP mI or vPIP mI-MR virus (1000 plaque-forming units per mouse). Lung tissues were collected at 6 d post-infection during acute infection. Compared with that of the WT or vPIP mI-MR viruses, lytic replication of the vPIP mI virus was highly attenuated (as much as the vPIP-S virus) in the lungs during acute infection (Figs. 6 ▸ *a* and 6 ▸ *b*). To further evaluate the latent infection in mice after intranasal inoculation, the spleen was harvested 17–18 d post-infection (Figs. 6 ▸ *c* and 6 ▸ *f*). Mice infected with vPIP mI or vPIP-S viruses had a smaller spleen in comparison with mice infected with WT or vPIP mI-MR viruses (Fig. 6 ▸ *c*). Just as in vPIP-S virus infection, vPIP mI virus infection showed attenuated viral latency in splenocytes as revealed by infectious-center assays and limiting-dilution assays (Figs. 6 ▸ *d* and 6 ▸ *e*). The viral genome loads in the splenocytes were lower in the mice infected with the vPIP mI or vPIP-S viruses than in mice infected with the WT or vPIP mI-MR viruses (Fig. 6 ▸ *f*). Taken together, these data suggest that the three N-terminal residues of vPIP (Phe5, Ser12 and Thr16) may be important for viral infection *in vivo* as well as for *in vitro* lytic replication.

### 3.7. The conserved mechanism of action of gammaherpesvirus *orf49*-encoded proteins   

**Figure 7:**
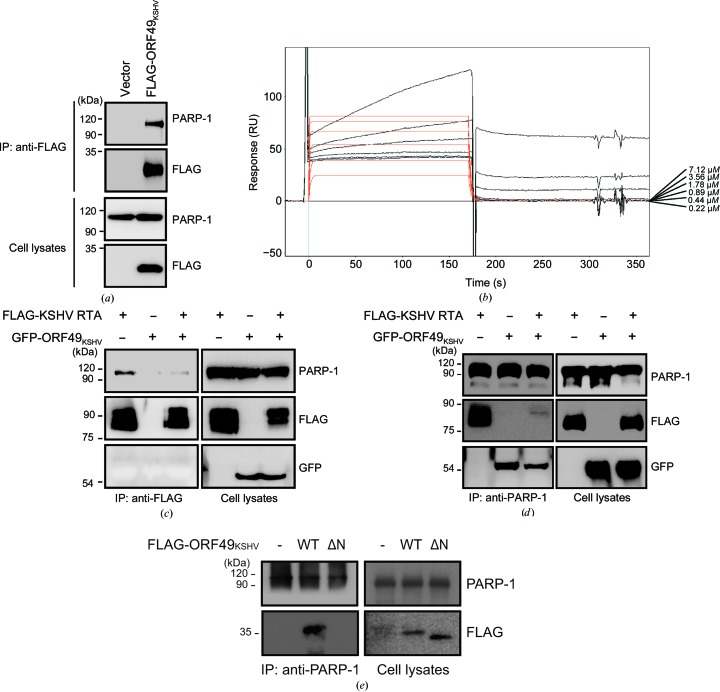
The conserved molecular mechanism of ORF49_KSHV_ interacting with PARP-1. (*a*) Interaction with PARP-1. FLAG-tagged ORF49_KSHV_ was transfected into HEK293T cells and incubated for 48 h. The cells were harvested and analyzed by co-IP assays with an anti-FLAG antibody. (*b*) SPR analysis of ORF49_KSHV_ with PARP-1. The ORF49_KSHV_ protein was injected at six concentrations (7.12, 3.56, 1.78, 0.89, 0.44 and 0.22 µ*M*). Dissociation data were collected for 120 s. The black lines show the actual data; the orange lines are curve fits. (*c*, *d*) Inhibition of interactions between RTA and PARP-1 by ORF49_KSHV_. GFP-tagged ORF49_KSHV_ was co-transfected with FLAG-tagged RTA into HEK293T cells. The cells were harvested 48 h post-transfection and subjected to co-IP assays with an anti-FLAG antibody (*c*) or an anti-PARP-1 antibody (*d*). The results were analyzed by Western blotting. (*e*) PARP-1 interaction of the ORF49_KSHV_ mutant. FLAG-tagged ORF49_KSHV_ or ORF49_KSHV_ ΔN was transfected into HEK293T cells. The cells were harvested 48 h post-transfection and assayed for PARP-1 interaction by co-IP assays with an anti-PARP-1 antibody. The results were analyzed by Western blotting.

KSHV, an oncogenic human gammaherpesvirus, also encodes ORF49 (ORF49_KSHV_), which can cooperate with RTA to activate lytic promoters (González *et al.*, 2006 ▸). We set out to determine whether gammaherpesvirus ORF49 homologs may share mechanisms of action (Fig. 7 ▸). Molecular interactions of ORF49_KSHV_ with PARP-1 were found in co-IP assays (Fig. 7 ▸ *a*). The SPR results indicated that ORF49_KSHV_ directly bound to PARP-1 with a *K*_d_ of 410 n*M* (Fig. 7 ▸ *b*). Moreover, reciprocal co-IP data showed that ORF49_KSHV_ interacted with PARP-1 and abrogated the interaction between KSHV RTA and PARP-1 (Figs. 7 ▸ *c* and 7 ▸ *d*), suggesting that ORF49_KSHV_ may share a conserved mechanism of action with MHV-68 vPIP in terms of derepressing RTA by sequestering PARP-1. In contrast to vPIP, however, a direct inter­action of ORF49_KSHV_ and RTA was not detected (Fig. 7 ▸ *c*). In addition, the results of SEC-MALS using purified ORF49_KSHV_ and co-IP assays suggested that ORF49_KSHV_ exists as a monomer in solution and in the cellular environment (Supplementary Fig. S4). Like vPIP, we also tested whether the functional importance of the N-terminus is conserved in ORF49_KSHV_. Alignment of the N-terminal regions of vPIP and ORF49_KSHV_ revealed limited homology between these proteins, with an extra 13 amino acids at the beginning of ORF49_KSHV_. However, they share the conserved structure of a short β-strand and an α-helix at the N-terminus. We constructed ORF49_KSHV_ ΔN (deletion of amino acids 2–36) lacking the N-terminal residues in a similar position to vPIP ΔN (Supplementary Fig. S5*a*). ORF49_KSHV_ and ORF49_KSHV_ ΔN were detected both in the nucleus and the cytoplasm (Supplementary Fig. S5*b*). However, unlike wild-type ORF49_KSHV_, the ORF49_KSHV_ ΔN mutant failed to interact with PARP-1 (Fig. 7 ▸ *e*). These results suggest that the functional importance of the N-terminus is conserved in both MHV-68 and KSHV gamma-2 herpesviruses. Despite the difference in dimerization and in interaction with RTA, our results clearly indicate that the ability of ORF49 homologs to interact with PARP-1 and interfere with the interactions between PARP-1 and RTA is highly conserved between these two oncogenic gamma-2 herpesviruses.

## 4. Discussion   

PARP-1, an abundant nuclear protein, participates in multiple cellular activities and is known to inhibit oncogenic gammaherpesvirus lytic replication by PARylating RTA, a key switch molecule in lytic replication, thus downregulating lytic genes (González *et al.*, 2006 ▸). ORF49 homologs are encoded by all gammaherpesviruses, and cooperate with RTA and positively regulate viral replication (González *et al.*, 2006 ▸; Hong *et al.*, 2004 ▸; Lee *et al.*, 2007 ▸). According to previous studies, MHV-68 ORF49 promotes viral lytic replication *via* interactions with PARP-1 and RTA by relieving the inhibitory effect of PARP-1 on RTA (Lee *et al.*, 2007 ▸; Noh *et al.*, 2012 ▸). Here, we present a structure-based molecular mechanism for a viral strategy using MHV-68 ORF49 to overcome PARP-1 inhibition during viral replication. MHV-68 ORF49 directly interacted with PARP-1 in solution without any other cellular factors; this action was found to be conserved in ORF49_KSHV_, thereby prompting us to propose a new name for MHV-68 ORF49: viral PARP-1-interacting protein (vPIP). The N-terminal β-strand region (Nβ) and α1 helix (amino-acid residues 2–26) turned out to be critical for the nuclear localization, PARP-1 interaction and molecular function of vPIP (Figs. 2 ▸, 3 ▸ and 4 ▸). Three residues at the N-terminus (Phe5, Ser12 and Thr16) were crucial for its PARP-1 interaction and molecular function. Furthermore, a recombinant virus harboring alanine substitutions of these three residues showed severely atten­uated viral replication both *in vitro* and *in vivo*, suggesting that the interaction of vPIP with PARP-1 is essential for the ability to facilitate viral replication.

In this study, direct interaction of vPIP with PARP-1 was confirmed *in vitro* in SPR assays, in addition to *in vivo* in co-IP assays (Fig. 1 ▸). Together with the previous study (Noh *et al.*, 2012 ▸), our results clearly identified PARP-1 as a genuine interaction partner and target of vPIP. ORF49_KSHV_ also strongly interacted with PARP-1 as shown in *in vivo* and *in vitro* assays (Fig. 7 ▸). Although interaction with RTA was not observed, ORF49_KSHV_ also exerted action on PARP-1, and this interaction abrogated the interaction of RTA and PARP-1. Therefore, these results suggest that the inter­action of *orf49*-encoded proteins with PARP-1 is conserved among gammaherpesviruses and is important for the function of these proteins in viral lytic replication.

According to its X-ray crystallographic structure, vPIP has a V-shaped conformation consisting of 12 α-helices and characteristic N-terminal β-strands (Nβ). Recently, the structure of ORF49_KSHV_ (PDB entry 5ipx) was reported to consist of 12 α-helices with two pseudo-domains (Hew *et al.*, 2017 ▸). The vPIP homologs of MHV-68 and KSHV share low sequence similarity (∼20%), with an r.m.s. deviation of 2.2 Å from *PyMOL* and a *Z* score of 24.5 from the *DALI* server (Holm & Laakso, 2016 ▸), but they have highly similar structural configurations of multiple helices, despite the introduction of unexpected mutations in the ORF49_KSHV_ structure (Q140P and Q179E; Hew *et al.*, 2017 ▸; Supplementary Fig. S3). These two proteins differ in that ORF49_KSHV_ has an N-terminus with an α-helix, whereas MHV-68 vPIP has an N-terminus with an Nβ (Supplementary Fig. S3). Moreover, although our structural and biochemical results indicate that MHV-68 vPIP forms a homodimer (Fig. 1 ▸), ORF49_KSHV_ does not exist as a dimer (Supplementary Fig. S4); this finding is consistent with a structural study of ORF49_KSHV_ (Hew *et al.*, 2017 ▸). Nonetheless, the results from vPIP mutants suggest that dimerization is not required for PARP-1 interaction and RTA derepression, as shown by vPIP ΔC: a mutant vPIP with intact function that does not form a dimer. Taken together, these results imply that homodimer formation by vPIP may be dispensable for vPIP function in RTA depression and PARP-1 interaction. Similar examples are seen in the cases of the translocation of ERK (Lidke *et al.*, 2010 ▸) and the modulation of the CaV2.2 channel by the 14-3-3 protein (Li *et al.*, 2007 ▸), where dimerization of ERK or 14-3-3 is dispensable for their function. However, it should be noted that vPIP dimerization may affect another function of vPIP which has not yet been elucidated.

A mutagenetic analyses based on the structural information revealed a critical domain and residues for vPIP function. Deletion of the N-terminus including the α1 helix and β-strands (*i.e.* creation of the mutant protein vPIP ΔN) switched the subcellular localization of vPIP to ‘cytoplasm only’ (from the typical location in both the nucleus and cytoplasm) and severely impaired the function of this protein. These results suggest that the N-terminus serves as a nuclear localization signal. Nevertheless, the sequence of the N-terminus does not show any known or predicted nuclear localization signal when analyzed by prediction programs such as *NucPred* and *Nuc-Ploc* (Brameier *et al.*, 2007 ▸; Shen & Chou, 2007 ▸). To examine whether the defective function of vPIP ΔN was owing to its exclusive localization in the cytoplasm, vPIP ΔN was forced to express in the nucleus by tagging a classical NLS from the SV40 T antigen. However, its function was still defective in interaction with PARP-1 and/or RTA 1 (Supplementary Fig. S2). These results suggest that the N-terminus not only serves as a nuclear localization signal but also plays a critical role in interacting with PARP-1, thereby interfering with the interaction between RTA and PARP-1. In contrast to vPIP ΔN, the vPIP mI mutant with alanine substitutions of Phe5, Ser12 and Thr16 is defective in function and in interaction with PARP-1, while maintaining an intact sub­cellular localization, suggesting that these three residues on the surface of vPIP are critical for its function and mechanism of action. The importance of these three residues was verified in the context of the viral genome, and it was found that the mutant virus vPIP mI shows weaker *in vitro* and *in vivo* replication, as does the vPIP-S virus, while the marker rescue virus (vPIP mI-MR) shows almost normal replication (Figs. 5 ▸ and 6 ▸). In addition to abated lytic replication, lower levels of latency in mice infected with the vPIP-S or vPIP mI virus were consistently observed; this effect may be owing to the inhibition of acute infection in the lungs (Fig. 6 ▸). Alternatively, this effect may be caused by the reduced ability of the vPIP-S or vPIP mI virus to establish latency in splenocytes or to efficiently reactivate from latently infected splenocytes. Nevertheless, these results highlight the importance of the inhibition of PARP-1 for viral fitness both *in vitro* and *in vivo*. To our knowledge, this is the first study to elucidate the molecular mechanism of action of any viral protein that regulates PARP-1 to promote viral replication.

In conclusion, we determined the X-ray crystallographic structure of vPIP, and structure-based mutagenesis experiments helped us to understand the viral strategy aimed at derepression of the inhibitory function of PARP-1 through a direct molecular interaction with vPIP, the mechanism of action of which is conserved between two oncogenic gammaherpesviruses.

## Supplementary Material

PDB reference: viral PARP-1-interacting protein, 6a4v

Supplementary Table and Figures.. DOI: 10.1107/S2052252518013854/lz5021sup1.pdf
