# Supplementary material for: Structure-based mechanism of action of a viral poly(ADP-ribose) polymerase 1-interacting protein facilitating virus replication
Source: IUCrJ. 2018 Oct 31;5(Pt 6):866–79. doi: 10.1107/S2052252518013854 (PMC6211522; doi:10.1107/S2052252518013854)
Supplement: Supplementary file 1 [file m-05-00866-sup1.pdf]

IUCrJ

Volume 6 (2019)

Supporting information for article:

Structure-based mechanism of action of a viral PARP-1-interacting protein facilitating virus replication

Woo-Chang Chung, Junsoo Kim, Byung Chul Kim, Hye-Ri Kang, JongHyeon Son, Hosam Ki, Kwang-Yeon Hwang and Moon Jung Song

Table S1 Primers used for molecular cloning of vPIP-related constructs.

Primer name	Primer sequence
vPIP for pET22b F	5’-GGGAATTCAATGGGGGATGAATTTTATTATCCTTCTCT-3’
vPIP for pET22b R	5’-GGCTCGAGCGAGGGAATTTCTGCAGCGATG-3’
vPIP WT F	5’-CCGCCGGAATTCATGGGGGATGAATTTTATTATCCTTC-3’
vPIP WT R	5’-CCGCTCGAGCTACGAGGGAATTTCTGCAGCG-3’
vPIP ΔN F	5’-CCGCCGGAATTCATGAGAGTAAGTGCCTGTTTG-3’
vPIP ΔN R	5’-CCGCTCGAGCTACGAGGGAATTTCTGCAGCG-3’
vPIP ΔC F	5’-CCGCCGGAATTCATGGGGGATGAATTTTATTATCCTTC-3’
vPIP ΔC R	5’-CCGCCGCTCGAGCTAAATTTGTAAACCACGCAG-3’
vPIP ΔN+NLS R	5’-CCG GCGGCCGC TTATACCTTTCTCTTCTTTTTTGG CGAGGGAATTTCTG-3’
ORF49$_{KSHV}$ ΔN F	5’-CCGGAATTCTACAATGGCCACCCTCAATCCAGA-3’
ORF49$_{KSHV}$ R	5’-GCGGGATCCTTATTGTATACTGAACAATGCG-3’
vPIP I F 1st	5’-CCGCCGCTCGAGCTAAATTTGTAAACCACGCAG-3’
vPIP I R1st	5’-CCGCCGCTCGAGCTAAATTTGTAAACCACGCAG-3’
vPIP I F 2nd	5’-CCGCCGGAATTCATGGGGGATGAATTTTATTATCCTTC-3’
vPIP I R 2nd	5’-CCGCTCGAGCTACGAGGGAATTTCTGCAGCG-3’
vPIP P F 1st	5’-CCGCCGGAATTCATGGGGGATGAATTTTATTATCCTTC-3’
vPIP P R 1st	5’-CAAAAATGGCTCTGTGGCCAAGTTGGCGCGCAGTGTCTGAC-3’
vPIP P F 2nd	5’-GTCAGACACTGCGCGCCAACTTGGCCACAGAGCCATTTTTG-3’
vPIP P R 2nd	5’-CCGCTCGAGCTACGAGGGAATTTCTGCAGCG-3’
vPIP I F1	5’-GCTTCCAGAGAAGGATAATAGGCCAGGAAGCCACGTTGTGTCTCAAAATC-3’
vPIP I F2	5’-CTATGACACAAAAGGCGTGGACCACGGCTTCCAGAGAAGGATAATAGGC-3’
vPIP I F3	5’-CACTTACTCTATTATGTTCCCTAGTATCTATGACACAAAAGGCGTGGACC-3’
vPIP R1	5’-CGTGGTCCACGCCTTTTGTGTCATAGATTACCCTGTTATCCCTATTTTCG-3’
vPIP R2	5’-GCCTATTATCCTTCTCTGGAAGCCGTGGTCCACGCCTTTTGTGTCATAG-3’
vPIP R3	5’-GAACAGAAAACATGGGGGATGAAGCCTATTATCCTTCTCTGGAAGC-3’
vPIP I-MR F1	5’-CTTTCCAGAGAAGGATAATAAAACAGGAAGCCACGTTGTGTCTCAAAATC-3’
vPIP I-MR F2	5’-CTATGACACAAAAGGTGTGGACCACGCTTTCCAGAGAAGGATAATAAAA-3’

vPIP I-MR F3	5’-CACTTACTCTATTATGTTCCCTAGTATCTATGACACAAAAGGTGTGGACCACG-3’
vPIP I-MR R1	5’-CGTGGTCCACACCTTTTGTGTCATAGATTACCCTGTTATCCCTATTTTCG-3’
vPIP I-MR R2	5’-TTTTATTATCCTTCTCTGGAAAGCGTGGTCCACACCTTTTGTGTCATAG-3’
vPIP I-MR R3	5’-GAACAGAAAACATGGGGGATGAATTTTATTATCCTTCTCTGGAAAG-3’

Table S2 Data collection and refinement statistics.

	Apo	SelMet-vPIP
Data Collection		
Wavelength (Å)	0.9792	0.9792
Space group	$P3_2\ 2\ 1$	$P3_2\ 2\ 1$
Unit-cell parameters (Å, °)	a = b = 134.179, c = 157.158	a = b =135.15, c = 158.44
	α = β = 90, γ = 120	α = β = 90, γ = 120
Resolution range (Å)	30 - 2.2 (2.24 - 2.2)	30 - 2.4 (2.44 -2.4)
Completeness (%)	90.5 (75.9)	92.2 (86.6)
Rsym† (%)	0.079 (0.414)	0.14 (5.48)
I/σ (I)	11.9 (1.6)	13.3 (0.3)
Redundancy	3.6 (1.8)	7.9 (6.2)
Total reflections	272254	636989
Refinement statistics		
Resolution range(Å)	29.8 - 2.2 (2.2 – 2.25)	
Unique reflections	44540	
R-work	23.6	
R-free	27.3	
RMS deviation		
Bond lengths, Å	0.01	
Bond angles, deg	1.84	
Ramachandran favored (%)	96.9	
Ramachandran outlier (%)	0.76	

Values in parentheses are for the highest resolution shell. $R_{\mathrm{sym}} = \sum_{hkli} | I_{hkli} - \langle I_{hkl} \rangle | / \sum_{hkli} I_{hkli}$, where *I* is the observed intensity, $\langle I \rangle$ is the average intensity, and *i* counts through all symmetry-related reflections.

Table S3 Kinetic constants measured by SPR.

Protein	Analyte	Ka ($M^{-1}s^{-1}$)	Kd(s^{-1})	KD (nM)
PARP1	vPIP	7.8(±6)x10^3	0.0073(±2)	930(±80)
PARP1	$ORF49_{KSHV}$	1.1(±2)x10^6	0.44(±9)	410(±30)

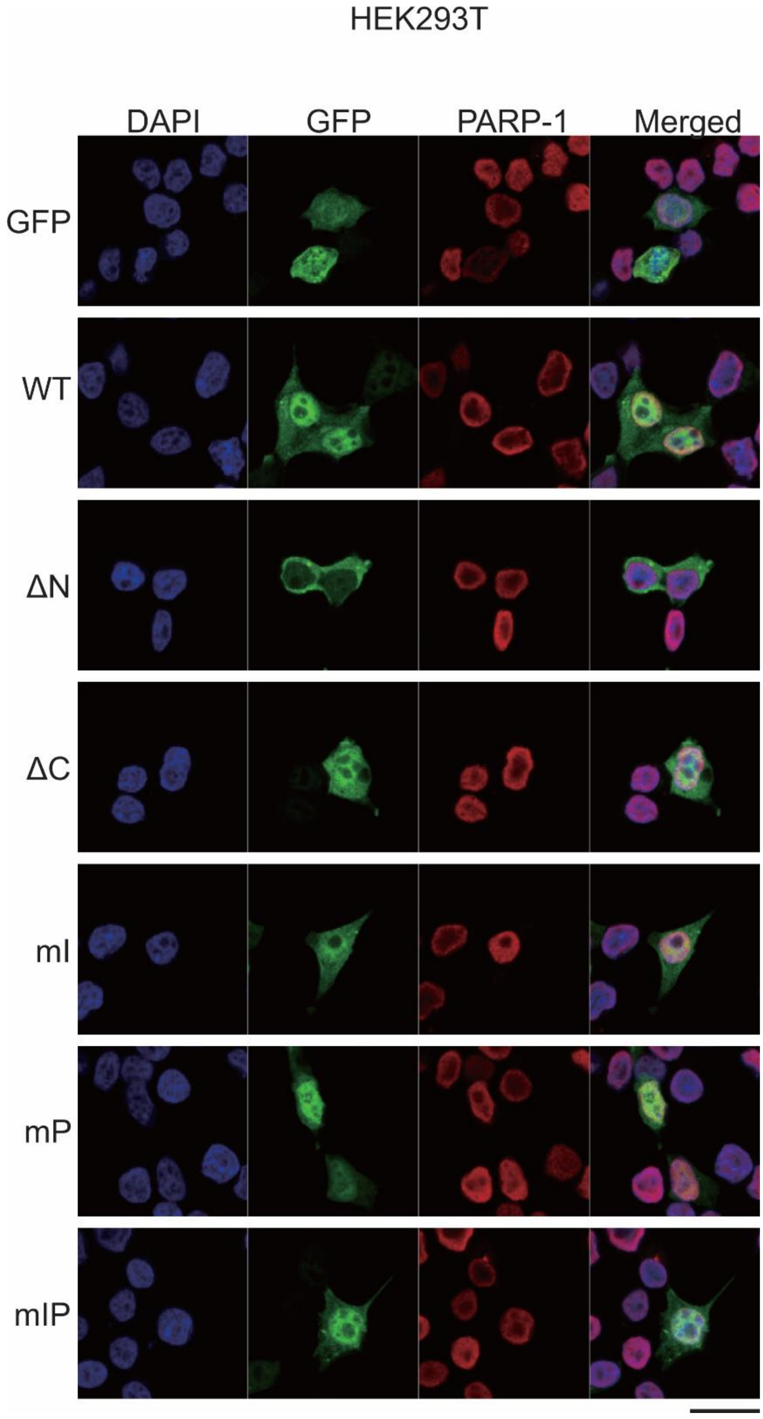

Figure S1 Subcellular localization of vPIP mutants in HEK293T cells. HEK293T cells were transfected with the GFP-tagged vPIP mutants, fixed at 24 h post-transfection, and immunostained with the anti-PARP-1 antibody. The nuclei were stained with DAPI (blue). Scale bar, 20 μm.

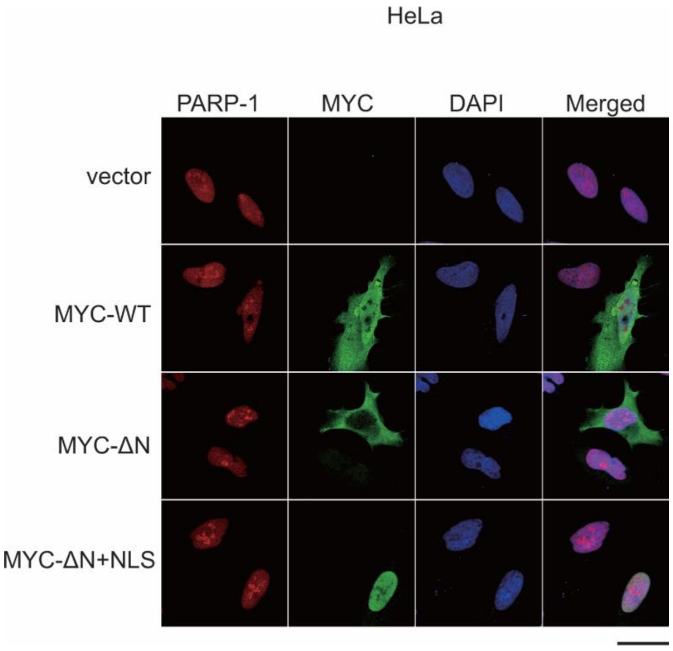

(*a*)

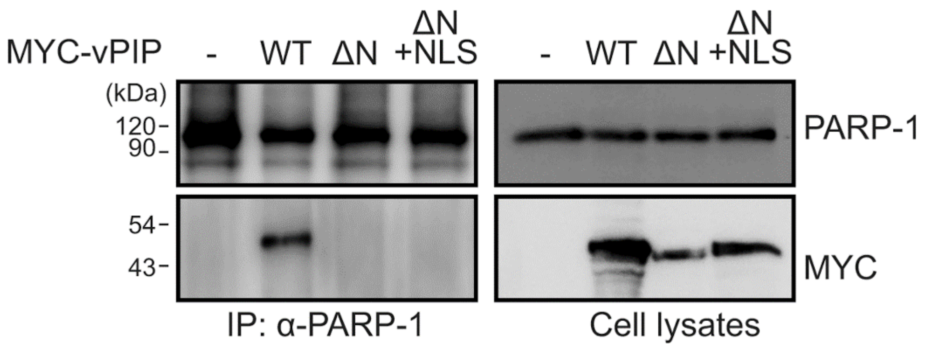

(*b*)

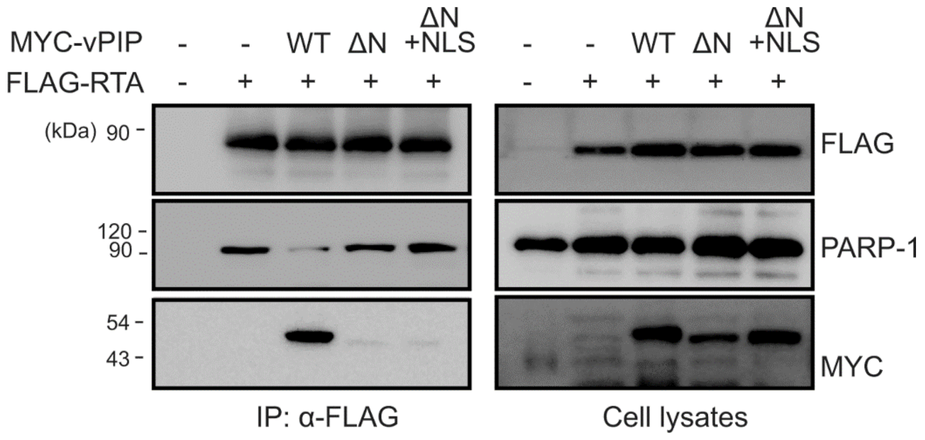

(*c*)

Figure S2 Characterization of vPIPΔN with NLS. (*a*) Subcellular localization of vPIP mutants. HeLa cells were transfected with the MYC-tagged vPIP WT, ΔN and ΔN with NLS. The nuclei were stained with DAPI (blue). Scale bar, 20 μm. (*b*) Interaction between vPIP mutants and PARP-1.

MYC-tagged vPIP mutants were transfected into HEK293T cells. The cells were harvested at 48 h post-transfection and analyzed by co-IP assays with the anti-PARP-1 antibody. The results were examined by western blotting. (*c*) Inhibition of interactions between RTA and PARP-1 by vPIP mutants. MYC-tagged vPIP mutants were cotransfected with FLAG-tagged RTA into HEK293T cells. The cells were harvested at 48 h post-transfection and subjected to coimmunoprecipitation (co-IP) assays with the anti-FLAG antibody. The results were examined by western blotting.

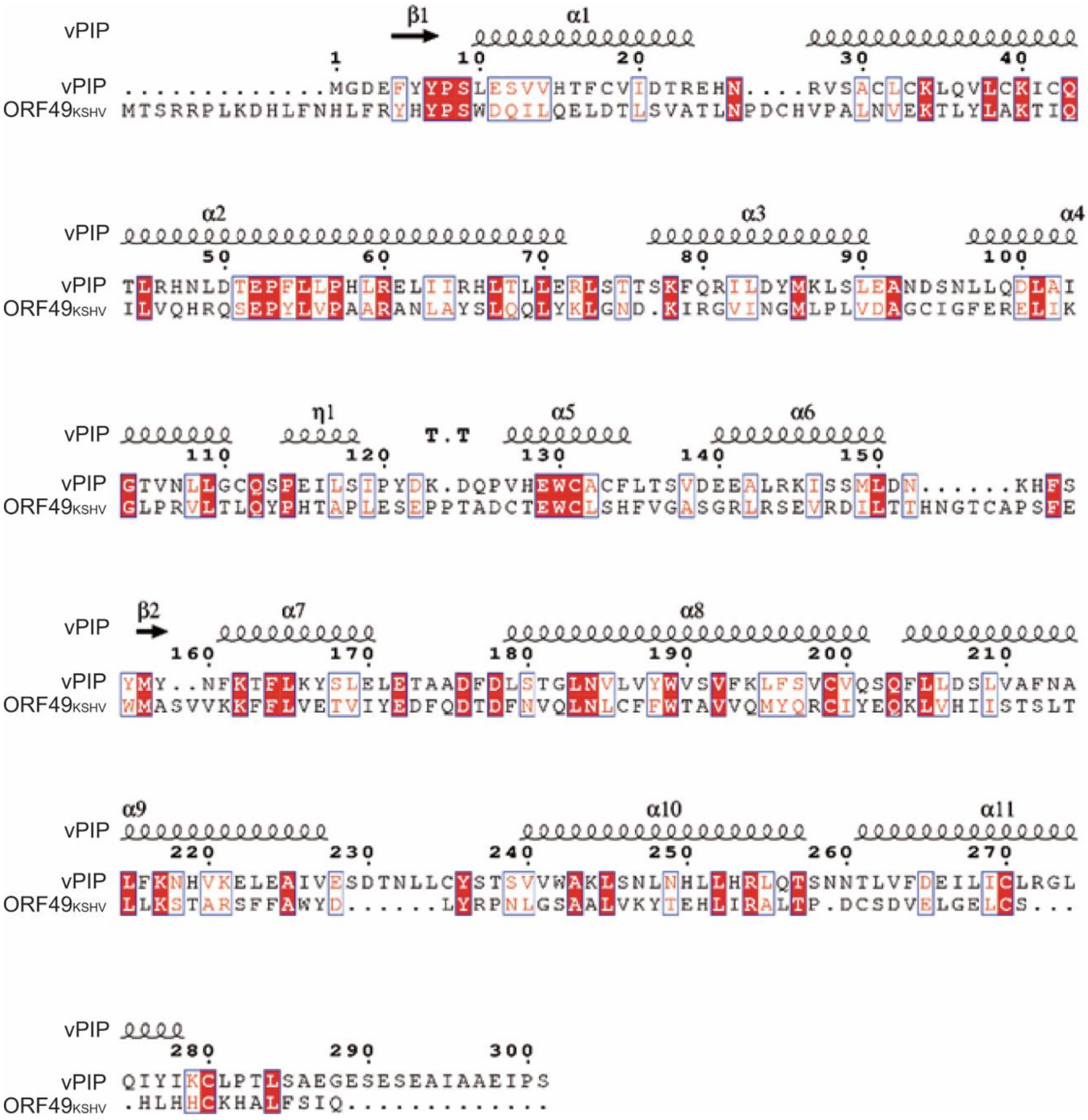

Figure S3 Sequence alignment between vPIP and $ORF49_{KSHV}$. The secondary structure of vPIP is shown for aa 1–280 of vPIP where structural information is resolved. The sequence alignment tool is ClustalW. The η symbol refers to a 310-helix, and strict β-turns are indicated as T.T. The diagram was built in ESPript (http://espript.ibcp.fr/ESPript/ESPript/).

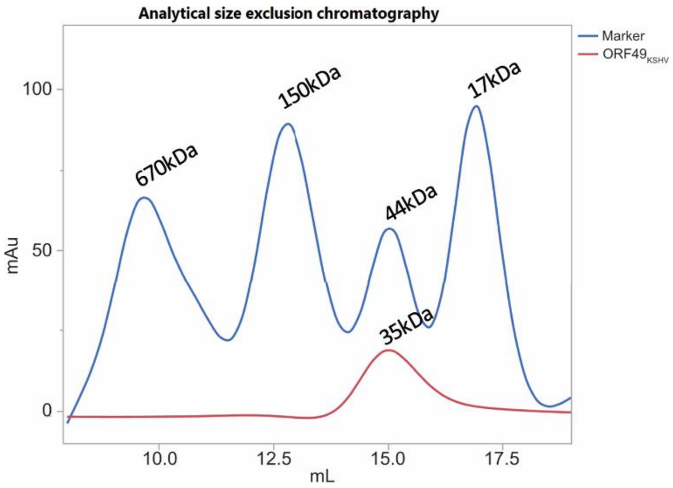
Analytical size exclusion chromatography
Marker
ORF49KSHV
670kDa
150kDa
44kDa
17kDa
35kDa
mAu
100
50
0
10.0
12.5
15.0
17.5
mL

(*a*)

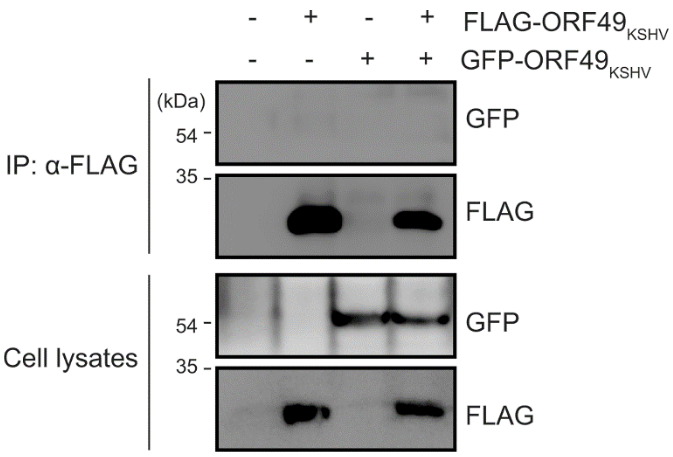
- + - + FLAG-ORF49KSHV
- - + + GFP-ORF49KSHV
(kDa)
54
35
GFP
FLAG
IP: α-FLAG
Cell lysates
54
35
GFP
FLAG

(*b*)

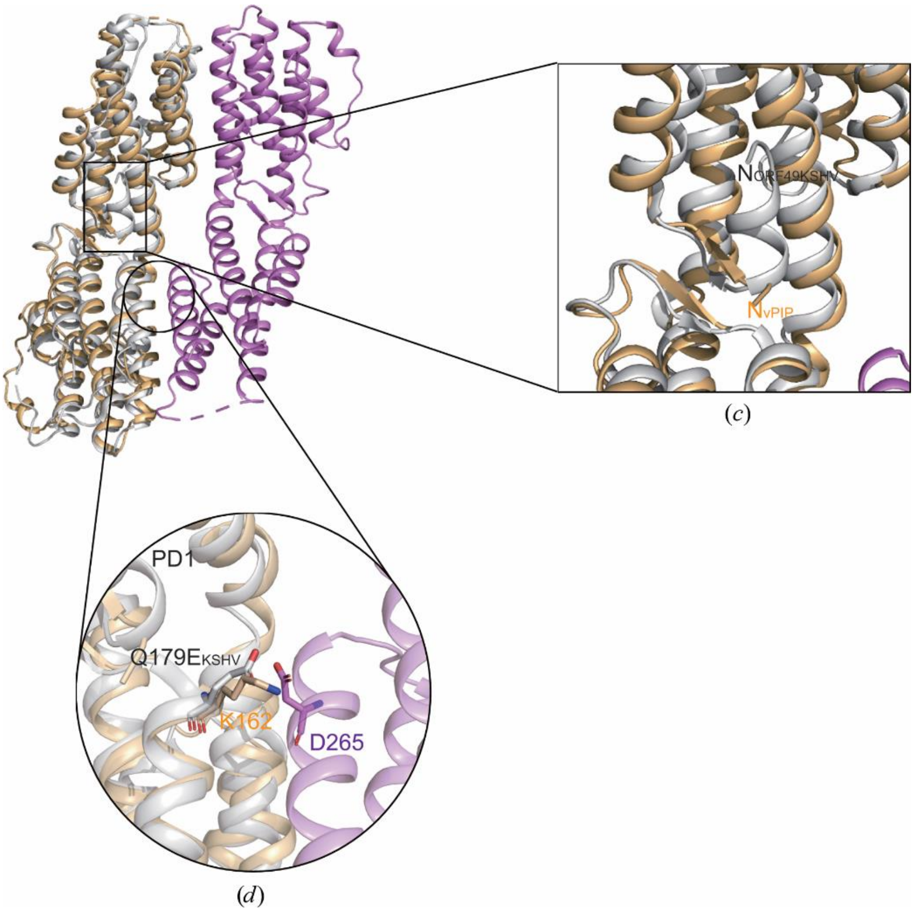

Figure S4 Unique features of ORF49$_{KSHV}$ in comparison with vPIP. (*a*) Multiple-angle light scattering (MALS) and refractive index curves for ORF49$_{KSHV}$. Light scattering (LS) is shown in blue, differential refractive index (dRI) is shown in red, and the molar mass is indicated. The buffer was removed and LS and refractive index were measured and plotted against a protein sample. (*b*) Failure of ORF49$_{KSHV}$ dimerization. GFP-tagged KSHV ORF49$_{KSHV}$ and FLAG-tagged ORF49$_{KSHV}$ were transfected into HEK293T cells for 48 h. The cells were harvested and subjected to co-IP assays with the anti-FLAG antibody. The results were analyzed by western blotting. (*c*) Superposition of vPIP and ORF49$_{KSHV}$. vPIP is light orange and violet and ORF49$_{KSHV}$ is gray. PD1, pseudo-domain 1. (*d*) A comparison between N-terminal regions of vPIP and ORF49$_{KSHV}$. vPIP is violet, and ORF49$_{KSHV}$ is gray.

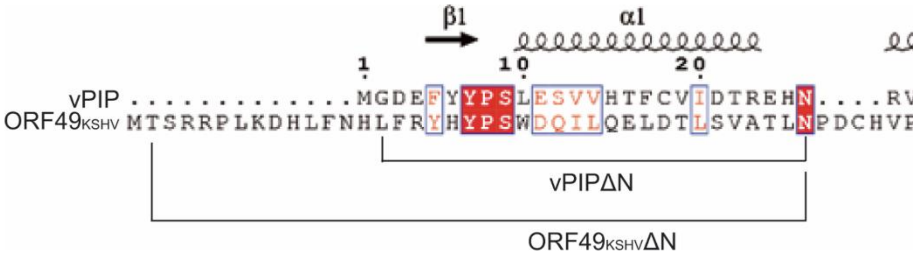

(*a*)

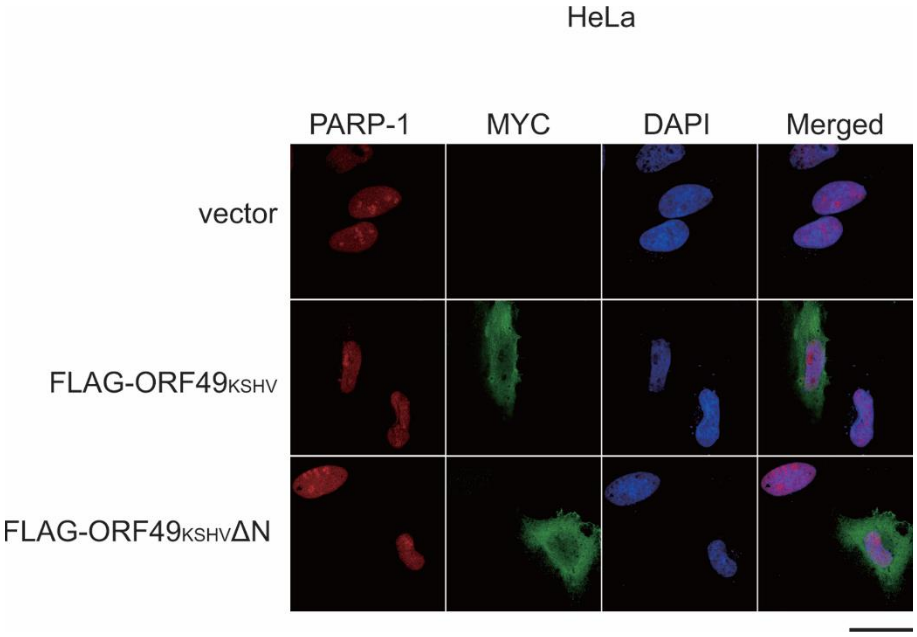

(*b*)

Figure S5 Characterization of a N-terminal deletion mutant of KSHV ORF49 (ORF49$_{KSHV}$ΔN). (*a*) Schematic diagram of the deleted N-terminus residues to construct ORF49$_{KSHV}$ΔN in comparison with vPIPΔN. The sequence alignment information is in Supplementary Fig. 3. (*b*) Subcellular localization of ORF49$_{KSHV}$ mutants. HeLa cells were transfected with the FLAG-tagged ORF49$_{KSHV}$ mutants, fixed at 24 h. The nuclei were stained with DAPI (blue). Scale bar, 20 μm.
